# Exploring causal relationship between 41 inflammatory cytokines and marginal zone lymphoma: A bidirectional Mendelian randomization study

**DOI:** 10.1515/med-2025-1171

**Published:** 2025-04-15

**Authors:** Xinhang Hu, Fenglei Yu, Muyun Peng, Zhi Yang, Yifan Ouyang, Zhe Zhang, Wangcheng Zhao, Xuyang Yi, Huali Hu, Xingchun Huang, Li Wang

**Affiliations:** Department of Thoracic Surgery, The Second Xiangya Hospital, Central South University, Changsha, 410000, China; Hunan Key Laboratory of Early Diagnosis and Precise Treatment of Lung Cancer, The Second Xiangya Hospital, Central South University, Changsha, 410000, China; Department of Thoracic Surgery, Hunan Rehabilitation Hospital, Changsha, 410000, China; Thoracic Surgery Research Laboratory, The Second Xiangya Hospital, Central South University, Changsha, 410000, China

**Keywords:** marginal zone lymphoma, inflammatory cytokines, Mendelian randomization, IL-10, MIG (CXCL9), B-NGF

## Abstract

**Purpose:**

Marginal zone lymphoma (MZL) is a rare subtype of non-Hodgkin lymphoma, and its diagnosis primarily relies on pathological biopsy. The study aims to investigate the causal relationships between 41 inflammatory cytokines and MZL using a two-sample bidirectional Mendelian randomization (MR) approach, providing new insights and methodologies for rapid differential diagnosis and treatment strategies.

**Methods:**

Causal associations between 41 inflammatory cytokines and MZL were examined using genetic variant data from two large-scale genome-wide association studies. The inverse variance weighting method was employed, and multiple sensitivity analyses, including MR-Egger, weighted median, simple model, and weighted model methods, were conducted to strengthen the robustness of the findings.

**Results:**

Elevated levels of MIG and IL-10 were associated with an increased risk of MZL (MIG: OR = 1.57, *p* = 0.035; IL-10: OR = 1.69, *p* = 0.021), while higher B-NGF levels exhibited a protective effect (OR = 0.46, *p* = 0.027). Reverse MR analysis revealed a negative correlation between MZL and IFN-γ levels (OR = 0.97, *p* = 0.015).

**Conclusions:**

MIG, IL-10, B-NGF, and IFN-γ are potential biomarkers and therapeutic targets for MZL. IFN-γ likely acts as a downstream molecule in MZL pathogenesis, offering novel insights into MZL-related research, clinical diagnosis, and treatment strategies.

## 1 Introduction

Marginal zone lymphoma (MZL) is an indolent subtype of non-Hodgkin B-cell lymphoma [1], comprising three related subtypes: mucosa-associated lymphoid tissue (MALT) lymphoma, nodal marginal zone B-cell lymphoma, and splenic MZL with or without villous lymphocytes [2–4]. There are two main clinical manifestations of MZL: extranodal and intranodal. Extranodal MALT lymphoma is the most common, accounting for 5–8% of all non-Hodgkin lymphomas, with the gastrointestinal tract being the most frequently affected site. Nodal and splenic MZL s are less common, each accounting for less than 1% of non-Hodgkin lymphoma cases [5]. With advances in medical science and technology, the diagnosis of MZL now typically involves a combination of clinical, morphological, immunophenotypic, and genetic criteria. However, a definitive diagnosis still often requires confirmation through pathological biopsy. Differentiating MZL from other tumors can be challenging. For example, MALT lymphoma has frequently been misdiagnosed as lung adenocarcinoma before pathological confirmation. Specific biomarkers for MZL, which could assist in its initial diagnosis, remain lacking [4–6]. Treatment for MZL traditionally includes surgical resection and chemotherapy [6–8]. Recent studies have demonstrated the effectiveness of anti-CD20 monoclonal antibodies (such as Rituximab) and CAR-T cell therapy in managing MZL [6,9,10]. The development of new drugs, including the covalent Bruton’s tyrosine kinase inhibitor ibrutinib [11], the B-cell lymphoma-2 inhibitor venetoclax [12], and phosphatidylinositol-3-kinase inhibitors [13] offers promising therapeutic options. However, no effective, clinically applicable targeted therapy strategies have been developed specifically for MZL-related biomarkers.

Although certain risk factors for extranodal MZL have been identified, such as autoimmune or chronic inflammatory diseases inducing MALT lymphoma [14] and *Helicobacter pylori* infection as a risk factor for gastric MALT lymphoma [15], the underlying etiology of MZL remains unclear. The role of inflammatory cytokines in cancer has garnered increasing attention recently. Inflammatory cytokines, which serve as important regulators, exhibit dual functions in cancer progression, from initiation to metastasis [16,17]. They can trigger immune responses that inhibit tumor growth, while also potentially promoting malignant processes such as cell transformation, growth, invasion, and metastasis [18]. In B-cell lymphomas, elevated levels of inflammatory cytokines such as IL-6, IL-10, VEGF, and IL-8 have been observed in patients with diffuse large B-cell lymphoma (DLBCL) [19,20]. However, these studies are primarily retrospective and subject to confounding factors, making it difficult to determine whether these cytokines have independent effects or to establish the direction and sequence of their interactions. Mendelian randomization (MR) offers a robust approach to address these limitations by assessing causality. Some research has suggested that MIG, PDGFbb, and MIP-1α may increase the risk of DLBCL, while IL-13 could serve as a protective factor [21,22]. The association between inflammatory cytokines and non-Hodgkin’s B-cell lymphoma has been reported exclusively in DLBCL, based on both retrospective analyses and MR-based studies. However, MZL, another subtype of non-Hodgkin’s B-cell lymphoma, has not been investigated for potential associations with inflammatory cytokines. Furthermore, no MR studies have confirmed a causal relationship between inflammatory cytokines and MZL. As a result, the potential role of inflammatory cytokines as biomarkers or therapeutic targets for the clinical diagnosis and treatment of MZL remains unclear. A two-sample bidirectional MR study was conducted to explore the causal relationship between inflammatory cytokines and MZL. The study aims to identify and validate biomarkers related to MZL and potential therapeutic targets within inflammatory cytokines, providing novel insights and strategies for MZL research, diagnosis, and treatment.

MR utilizes single nucleotide polymorphisms (SNPs) as genetic instrumental variables (IVs), offering a unique approach to establish causal relationships between exposures and clinical outcomes by leveraging genetic variation in nonexperimental data. MR effectively addresses confounding factors and minimizes reverse-causation bias [23,24]. Unlike traditional observational studies, MR benefits from the random allocation of alleles during meiosis, providing stronger causal inferences and more precise determination of causal directions [25,26]. Increasing evidence suggests that human genetic data related to inflammatory cytokine levels can offer valuable insights for clinical research [21]. Consequently, MR has emerged as a robust approach for investigating the causal role of inflammatory cytokines in the development of MZL. In this study, we conducted a two-sample bidirectional MR. First, we selected suitable genetic instruments for 41 inflammatory cytokines using genome-wide association study (GWAS) data. Then, we explored the association between these cytokines and MZL, followed by reverse MR analysis to assess causality direction. This study aims to identify potential biomarkers and therapeutic targets for MZL, providing a novel approach to its clinical diagnosis and treatment, while also offering fresh insights into the pathogenic factors and mechanisms underlying MZL.

## 2 Methods

### 2.1 MR hypothesis

**Figure 1:**
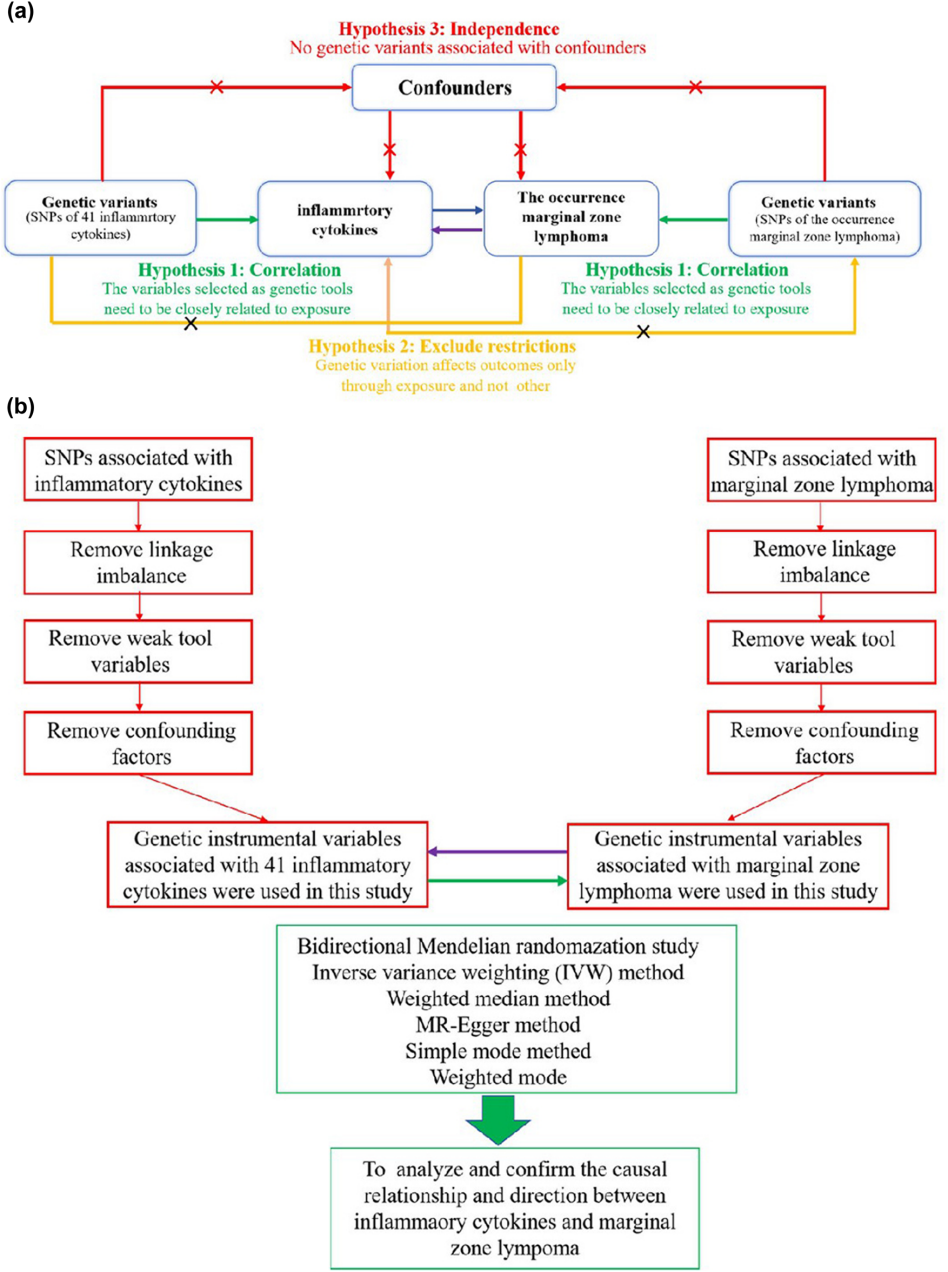
Bidirectional two-sample MR study structure. (a) The diagram outlines the foundational principles for MR analysis, emphasizing the key assumptions: (1) correlation, genetic variants are significantly associated with the exposure; (2) independence, genetic instruments should not be related to confounders; (3) exclusion restrictions, genetic variants affect the outcome only through the exposure, without alternative pathways. (b) The conceptual design and analysis approach of the bidirectional MR used in this study.

Figure 1 illustrates the conceptual framework of a two-sample bidirectional MR study. The analysis relies on three key assumptions: correlation, independence, and exclusion restrictions. The correlation assumption requires a strong association between the genetic variant used as an IV and the exposure. Independence ensures that the genetic variant is not influenced by confounding factors. Exclusion restrictions state that the genetic variant affects the outcome only through its influence on the exposure, without any alternative pathways of effect [27]. In this study, significant SNPs related to 41 inflammatory cytokines and MZL were selected from data obtained from two previously published GWAS. Initially, genetic instruments for each cytokine were identified to explore their causal effects on MZL. Subsequently, genetic IVs associated with MZL were used to infer causality between MZL and each inflammatory cytokine, aiming to clarify the upstream and downstream causal relationships between these cytokines and MZL.

### 2.2 Data sources

The datasets used in this analysis were obtained from publicly available GWAS summary data, negating the need for additional ethical approval. Data on 41 inflammatory cytokines were retrieved by reanalyzing GWAS results related to serum cytokines [28], available at https://doi.org/10.5523/bris.3g3i5smgghp0s2uvm1doflkx9x. Data on MZL were extracted from C3_MARGINAL_ZONE_LYMPHOMA_EXALLC dataset in the R11_manifest Finnish database, which included 256 cases of European origin and 345,118 healthy controls. All cases were diagnosed through pathological examination. The FinnGen project, launched in 2017 in Finland, integrates genetic data from the Finnish Biobank with disease outcomes from the Finnish Health Registry, enabling the study of genotype–phenotype associations in the Finnish population. Genotyping of FinnGen participants was performed using Illumina and Affymetrix chip arrays (Illumina Inc., San Diego and Thermo Fisher Scientific, Santa Clara, CA, USA). Imputation was conducted with 3,775 genome-wide SISu v3 imputation reference panels, resulting in 16,962,023 variants. Stringent quality control measures were applied to exclude individuals with unclear sex, high deletion rates (>5%), abnormal heterozygosity, or non-Finnish ancestry. Variants with deletion rates exceeding 2%, low Hardy–Weinberg equilibrium *p*-values (<10⁻⁶), or minor allele counts below 3 were also excluded [25]. The exposure and outcome data used in the two-sample MR analysis were derived from separate databases and research projects, ensuring no overlap of study participants.

### 2.3 Selection of genetic IVs

A rigorous quality control process was applied to ensure that the chosen IVs satisfied the key assumptions of MR analysis: (1) a strong association between genetic variants and the exposure, (2) independence from confounding factors, and (3) the IVs affecting the outcome exclusively through the exposure. Initially, SNPs strongly associated with MZL and inflammatory cytokines were selected using a significance threshold of *p* < 5 × 10⁻⁸. Given the limited number of SNPs meeting this threshold for some exposures, the threshold was adjusted to *p* < 5 × 10⁻⁶ for forward exposure factors and *p* < 5 × 10⁻⁵ for reverse exposure factors. To minimize linkage disequilibrium (LD) effects, we applied a threshold of *r* ^2^ < 0.001 for LD, with a genetic distance of 10,000 kb [29]. Next, we calculated the proportion of variance explained by each SNP using the following formula for *R* ^2^: *R* ^2^ = (2 × EAF × (1 − EAF) × Beta^2^)/[(2 × EAF × (1 − EAF) × Beta^2^) + (2 × EAF × (1 − EAF) × *N* × SE^2^)] [30]. The *F*-statistic was computed to assess the potential for weak IV bias using the formula: *F* = *R* ^2^(*N* − *K* − 1)/*K*(1 − *R* ^2^). An *F*-statistic greater than 10 indicated no weak instrument bias, and data meeting this criterion were retained. Furthermore, we used the Phenoscanner database (http://www.phenoscanner.medschl.cam.ac.uk/) to screen SNPs and exclude those associated with confounding factors or the outcome (*P* < 5 × 10⁻⁸), reducing the risk of pleiotropy. The SNPs that passed this screening were selected as genetic IVs for the analysis [31].

### 2.4 MR analysis

Data analysis was conducted using R 4.3.2 software, with the “TwosampleMR” and “MR-PRESSO” packages employed to ensure a comprehensive and rigorous MR approach.

The primary analytical method utilized was the classical inverse variance weighting (IVW) method, known for its efficiency and strong statistical power, even in the presence of heterogeneity [32]. Weighted median, MR-Egger method, simple mode, and weighted mode approaches were utilized to perform supplementary auxiliary analyses to ensure the confidence of causal relationship. MR-Egger regression can detect and adjust for pleiotropy, although it produces estimates with lower precision [33]. The weighted median method provides accurate estimates if at least 50% of the IVs are valid [34]. The simple mode method, while less powerful than IVW, offers robustness against pleiotropy [35], and weighted mode estimation is sensitive to bandwidth choices, making it more challenging [36]. Sensitivity analyses were also performed utilizing MR-Egger regression, which incorporates an additional intercept term (*α*) to test for directional pleiotropy, assuming that the direct effect of a genetic variant on the outcome (not through exposure) is independent of its effect on the exposure [33]. The MR-PRESSO method was used to detect and remove outlier SNPs that could introduce bias due to confounding variables. Heterogeneity among SNPs was assessed using Cochran’s *Q* test. A *Q* value larger than the number of instruments minus one indicates heterogeneity and invalid instruments, while a *Q* statistic with a *p-*value <0.05 suggests the presence of heterogeneity. Given that heterogeneity may violate the assumptions of MR and compromise the reliability of results, datasets exhibiting heterogeneity were excluded [37–40]. The IVW method, grounded in conventional meta-analysis principles, offers a robust statistical foundation, enhancing result reliability. Its superior statistical efficiency, power, applicability, and transparency make it a preferred methodology. Thus, significant results (*p* < 0.05) from IVW analysis were considered reliable, even if supplementary methods like weighted or simple modes did not yield significant outcomes (*p* < 0.05), as long as the direction of the *β*-value remained consistent and there were no signs of horizontal pleiotropy, confounding bias, or heterogeneity. The MR-Egger method was employed to address potential horizontal pleiotropy, and no heterogeneity was found. In cases of heterogeneity, the weighted median and random-effects IVW methods were used, provided that no polymorphisms were detected [41–46]. Additionally, leave-one-out analyses were conducted to determine whether any single SNP influenced the causal estimates, further ensuring the robustness of the findings [47].


**Ethical approval:** The datasets utilized in this analysis were derived from publicly available genome-wide association study (9GWAS) summary data, eliminating the need for additional ethical approval.

## 3 Results

### 3.1 Genetic predictors related to 41 circulating inflammatory factors and MZL

To ensure an adequate number of SNPs for MR analysis, a significance threshold of *p* < 5 × 10⁻⁶ was applied for SNPs associated with the 41 inflammatory cytokines, and a threshold of *p* < 5 × 10⁻⁵ was set for SNPs associated with MZL. After filtering out SNPs exhibiting LD, weak IVs, and those related to confounding factors, a total of 456 SNPs associated with inflammatory cytokines were identified, with *F*-statistic values ranging from 20.77 to 782.3. Additionally, 13 MZL-related SNPs were identified, with *F*-statistic values ranging from 19.57 to 34.45. All IVs had *F*-statistics above 10, indicating no weak instrument bias. Detailed information is provided in Tables S1 and S2.

### 3.2 Effects of 41 inflammatory cytokines on the occurrence of MZL

**Figure 2:**
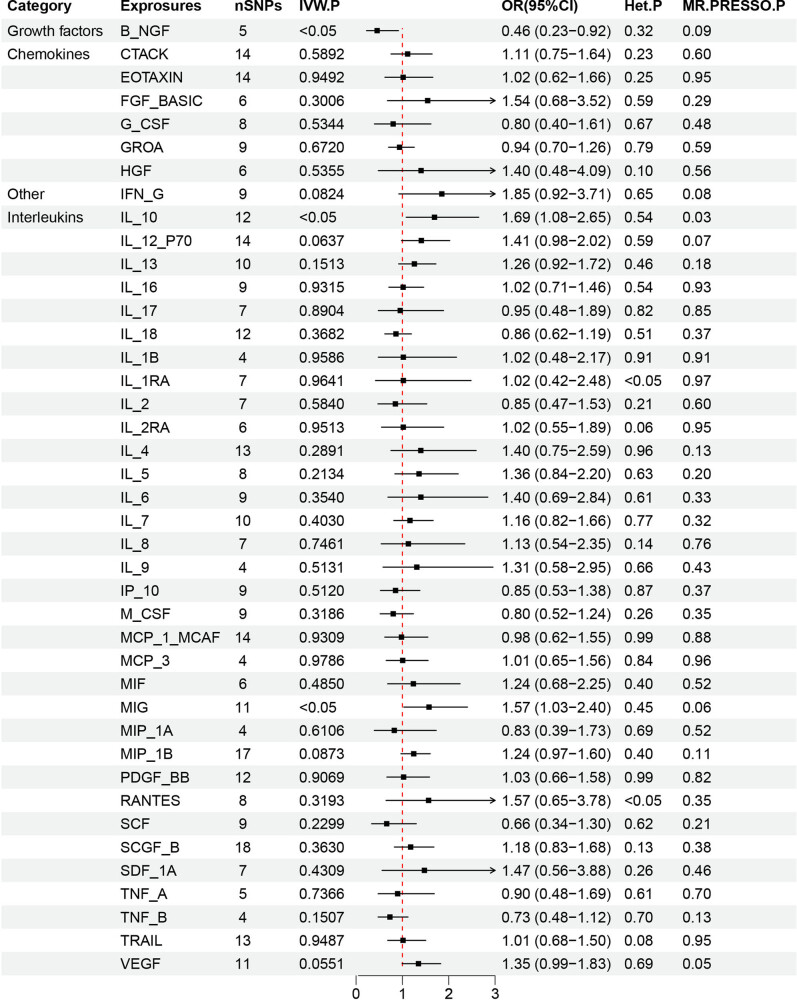
Forest plot of forward MR results for 41 inflammatory cytokines associated with MZL. This forest plot presents the results of IVW analysis to assess the potential causal relationship between 41 inflammatory cytokines as the exposure factors and the risk of MZL as the outcome. Using a significance threshold of *P* < 0.05, the plot reveals that higher genetically predicted levels of MIG and IL-10 are positively correlated with an increased likelihood of MZL (MIG: OR = 1.57, 95% CI = 1.03–2.40, *p* = 0.035; IL-10: OR = 1.69, 95% CI = 1.08–2.65, *p* = 0.021). In contrast, higher genetically predicted levels of B-NGF are negatively associated with the likelihood of MZL (OR = 0.46, 95% CI = 0.23–0.92, *p* = 0.027). The heterogeneity test showed no evidence of heterogeneity (MIG: *p* = 0.45; IL-10: *p* = 0.54; B-NGF: *p* = 0.32).

**Figure 3:**
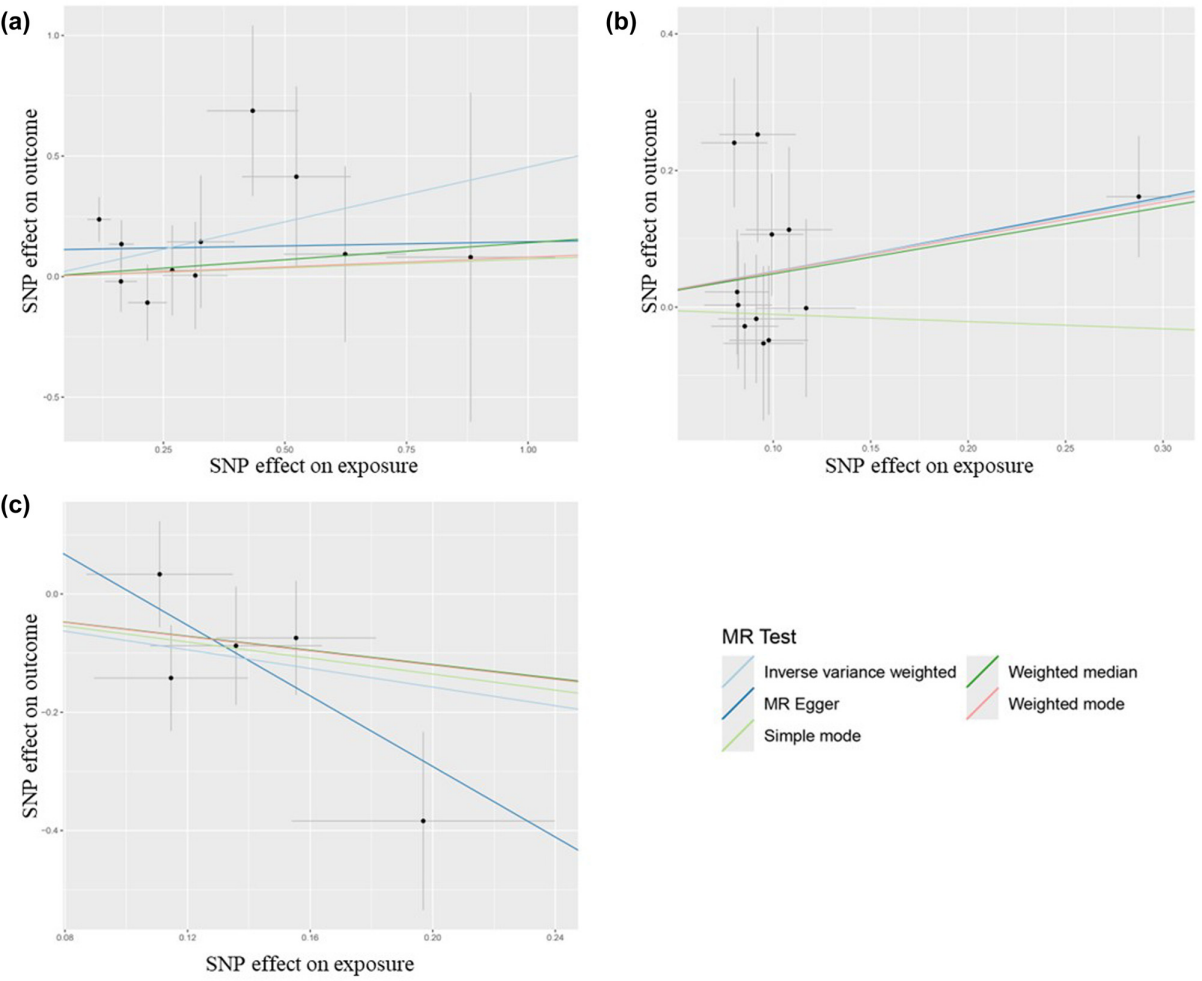
Scatter plot for positive MR analysis of MIG, IL-10, B-NGF, and MZL. (a) Funnel plot of forward MR analysis of the effects of MIG on MZL. (b) Funnel plot of forward MR analysis of the effects of IL-10 on MZL. (c) Funnel plot of forward MR analysis of the effects of B-NGF on MZL.

**Figure 4:**
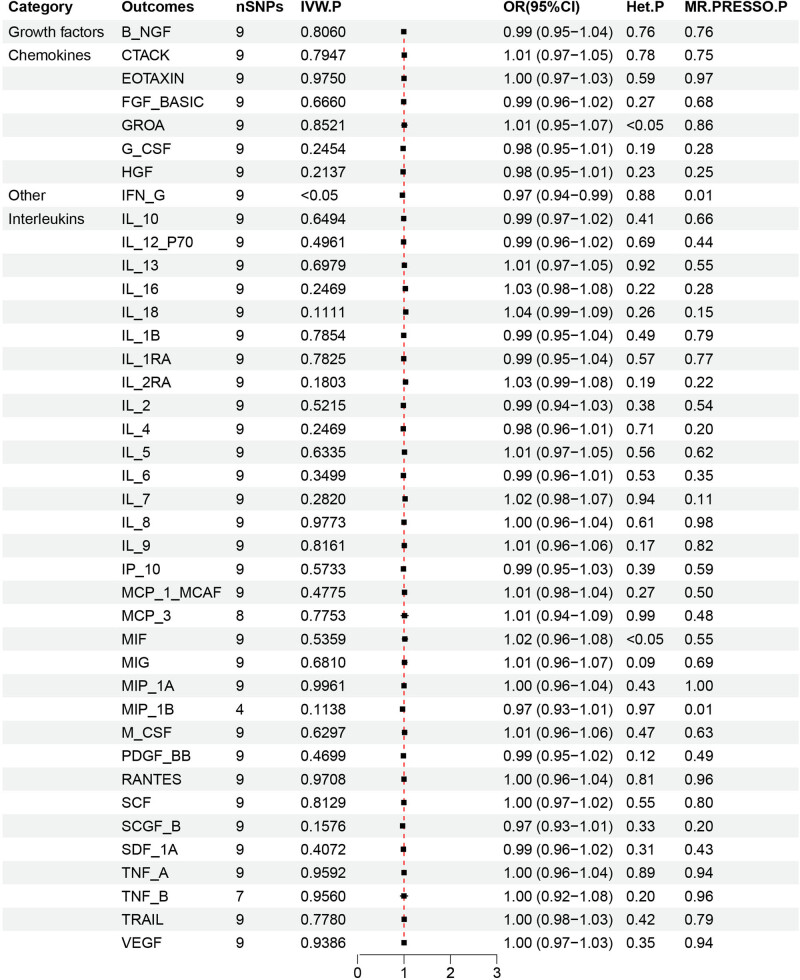
Forest plot of reverse MR data for 41 inflammatory cytokines associated with MZL. This forest plot highlights the use of IVW analysis to explore the potential causal relationship between the risk of MZL as the exposure factor and the levels of inflammatory cytokines as the outcome. At a significance threshold of *P* < 0.05, the results demonstrated that an increased likelihood of MZL was inversely associated with IFN-γ levels (OR = 0.97, 95% CI = 0.94–0.99, *p* = 0.015). The strength of this evidence was further supported by MR-PRESSO analysis (*p* = 0.01). The heterogeneity test showed no evidence of heterogeneity (*p* = 0.88).

The results of the forward MR analysis are presented in Figure 2 and further detailed in Table S3. This analysis assessed 41 inflammatory cytokines as exposure factors and MZL as the outcome. The primary focus was determining whether a positive causal relationship exists between each cytokine and the increased risk of MZL, with statistical significance indicated by a *p*-value <0.05 derived from the IVW) method. Additionally, the odds ratio (OR) value was used to assess whether the exposure factor acts as a risk or protective factor (OR > 1 suggests a risk factor, while OR < 1 indicates a protective factor). The *p*-value from the *Q* test was employed to evaluate heterogeneity (*p* < 0.05 suggests the presence of heterogeneity, while *p* > 0.05 suggests no heterogeneity). The IVW analysis revealed that genetically predicted higher levels of MIG and IL-10 were significantly associated with an increased risk of MZL (MIG: OR = 1.57, 95% CI = 1.03–2.40, *p* = 0.035; IL-10: OR = 1.69, 95% CI = 1.08–2.65, *p* = 0.021). In contrast, higher genetically predicted levels of B-NGF were associated with a decreased likelihood of MZL (OR = 0.46, 95% CI = 0.23–0.92, *p* = 0.027). The visualization results are shown in Figures 3 and S1–S3. The MR-Egger analysis showed no evidence of pleiotropy for MIG (*p* = 0.30), IL-10 (*p* = 0.96), or B-NGF (*p* = 0.29). The heterogeneity test also indicated no significant heterogeneity (MIG: *p* = 0.45; IL-10: *p* = 0.54; B-NGF: *p* = 0.32). MR-PRESSO analysis did not identify any outliers among the SNPs for the 41 inflammatory cytokines. The robustness of these findings was further confirmed through leave-one-out analysis, with *β* values from MR-Egger, weighted median, simple mode, and weighted mode analyses consistent with the IVW results. No other inflammatory cytokines were found to have a significant association with the development of MZL (Figure 4).

### 3.3 Effects of MZL on 41 inflammatory cytokines

**Figure 5:**
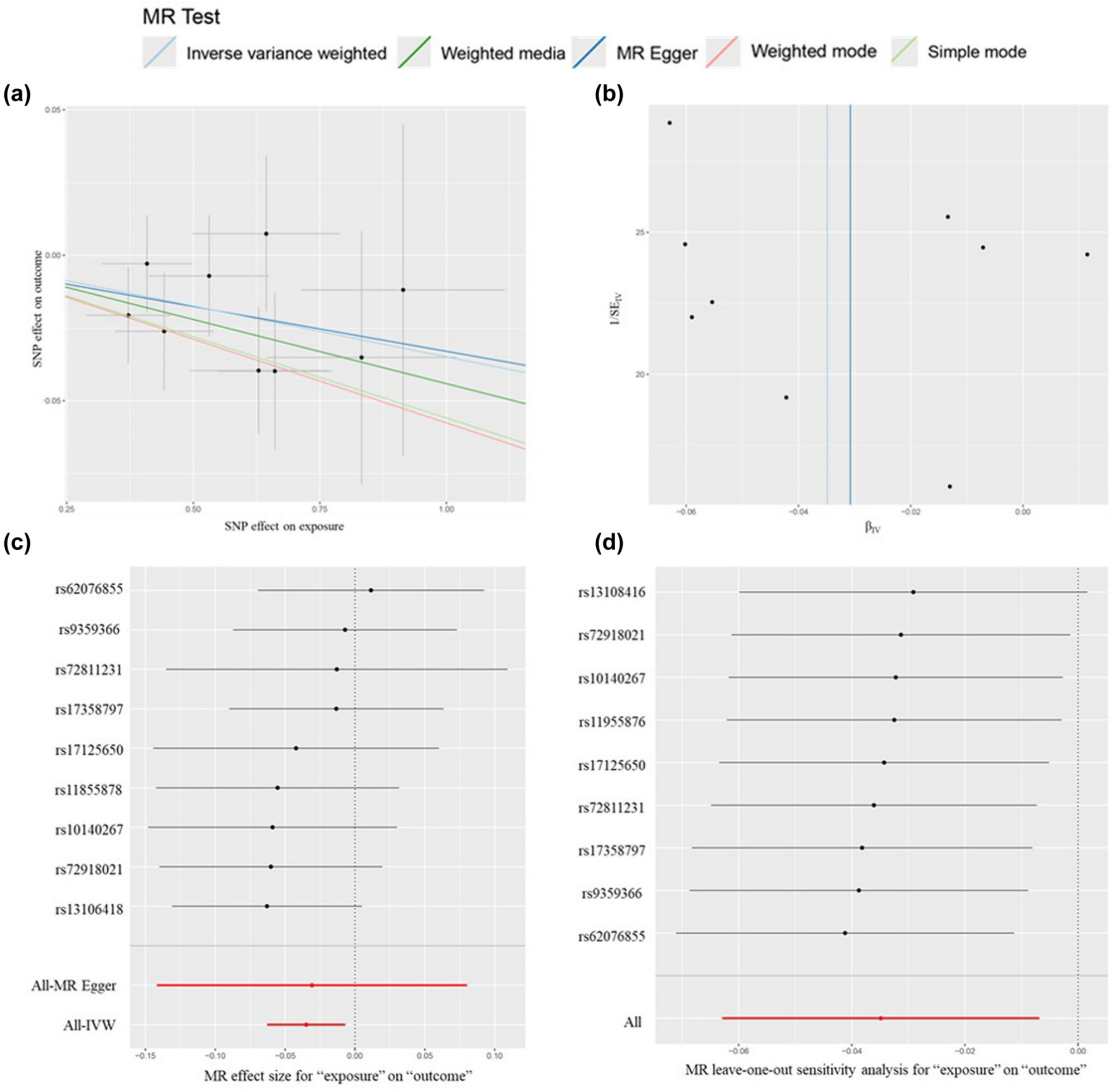
Visual results for reverse MR analysis of IFN-G and MZL. (a) Scatter plot of reverse MR analysis of IFN-G and MZL. (b) Funnel plot of reverse MR analysis of IFN-G and MZL. (c) Forest plot of reverse MR analysis of IFN-G and MZL. (d) Leave-one-out analyses of reverse MR analysis of IFN-G and MZL.

A reverse MR analysis was conducted to provide additional evidence and clarify the causal direction between inflammatory cytokines and MZL. MZL was used as the exposure factor, with the 41 inflammatory cytokines as the outcomes. The analysis focused on evaluating the causal relationship between each cytokine and MZL, specifically assessing whether increased levels of cytokines were associated with a higher risk of MZL. Causal relationships were determined based on *p*-values from the IVW method, with *p* < 0.05 indicating statistical significance. The OR was used to assess whether each cytokine acted as a risk or protective factor, where OR > 1 indicates a risk factor, and OR < 1 indicates a protective factor. The *p*-value from the *Q* test was also used to assess heterogeneity, with *p* < 0.05 indicating heterogeneity and *p* > 0.05 suggesting no significant heterogeneity. The IVW analysis found that higher levels of IFN-G were inversely associated with MZL risk (OR = 0.97, 95% CI = 0.94–0.99, *p* = 0.015). No horizontal pleiotropy was detected for IFN-G (*p* = 0.94) in the MR-Egger analysis, and no heterogeneity was observed (*p* = 0.88). MR-PRESSO analysis also identified no outlier SNPs among the exposure factors. The robustness of these findings was confirmed through leave-one-out analysis, with *β* values from MR-Egger, simple mode, weighted median, and weighted mode methods consistent with the IVW results. Figure 5 illustrates the causal relationship between MZL and changes in IFN-γ levels. No significant evidence was found to suggest that MZL influences the levels of other inflammatory cytokines.

## 4 Discussion

This study aimed to investigate the causal relationship between 41 inflammatory cytokines and MZL using a two-sample bidirectional MR approach to elucidate the directionality of this relationship and their roles in MZL development. Our results suggest that monokine induced by gamma interferon (MIG) and interleukin-10 (IL-10) may act as risk factors for MZL, while brain-derived neurotrophic factor (B-NGF) could serve as a protective factor against MZL Reverse MR analysis excluded MIG, IL-10, and B-NGF as downstream molecules of MZL. Furthermore, when MZL was considered an exposure factor, a decrease in interferon-gamma (IFN-G) levels was observed, highlighting potential pathogenic pathways and molecular mechanisms. These findings offer valuable insights and provide a clinical foundation for early diagnosis and further investigation into the pathogenesis of MZL.

MIG, located on human chromosome 4 [48], is involved in leukocyte chemotaxis, differentiation, proliferation, and tissue migration [49]. Induced by IFN-γ, MIG acts as a chemoattractant for T cells, with elevated circulating levels serving as markers of the host immune response, particularly in Th1 cells [50]. The role of MIG in the lymphoma microenvironment, particularly its involvement in B-cell lymphoma development, has been highlighted in several studies. Yu et al. [22] identified MIG as a contributing risk factor for DLBCL. Zhou et al. [51] found significantly higher MIG levels in DLBCL tissues compared to normal tissues. However, most research on MIG and lymphoma has focused on DLBCL. Jiang et al. [21] proposed that MIG may increase DLBCL risk by influencing the gut microbiome, particularly the genus *Ruminococcus* UCG-002. Ruiduo et al. [52] demonstrated that MIG promotes DLBCL progression via β-catenin upregulation. Despite these findings, there is limited research on MIG’s involvement in MZL. To date, no studies have explored the relationship between MIG and MZL or their interaction mechanisms. Our study addresses this gap by providing direct evidence of a causal relationship between MIG and MZL. However, the precise molecular mechanisms by which MIG influences MZL remain unclear, and further experimental studies are needed to investigate whether MIG affects MZL through interactions with the intestinal microbiota or β-catenin regulation.

IL-10 produced by various immune cells and encoded by the IL-10 gene on chromosome 1q32, is crucial in regulating the inflammatory response [53]. IL-10 exerts a dual regulatory effect on the immune system, promoting immune responses through CD8^+^ T cells while negatively regulating them via regulatory T cells [54]. Most studies on IL-10 in B-cell lymphomas have focused on DLBCL, consistently linking high IL-10 expression with poor prognosis. Shen et al. [19] and Bao et al. [55] found that IL-10 is highly expressed in DLBCL and associated with poor prognosis. Wu et al. [56] and Mishina et al. [57] recognized IL-10 as a factor that increases the risk of DLBCL and proposed it as a potential biomarker for diagnosing. Stirm et al. further explored the biological mechanisms of IL-10 in promoting DLBCL growth, suggesting that IL-10 induces tumor immune evasion, thereby driving progression [58]. However, most research on IL-10 in B-cell lymphoma has concentrated on DLBCL, with limited studies examining the relationship between IL-10 and MZL, another form of non-Hodgkin B-cell lymphoma. A recent study proposed IL-10 as a risk factor for gastric MALT lymphoma [59], a subtype of MZL, which supports the hypothesis that IL-10 plays a significant role in MZL development. This finding provides a theoretical foundation for further exploration of how IL-10 influences MZL pathogenesis, particularly in relation to tumor immune escape mechanisms.

B-NGF, a key nerve growth factor (NGF) family member, shares biological functions with NGF. As an essential neurotrophic factor, NGF is crucial for the survival and differentiation of neurons, significantly contributing to the growth and maintenance of the nervous system [60]. However, research on the relationship between B-NGF and tumors is limited. Existing studies suggest that NGF and B-NGF jointly participate in angiogenesis, promoting cancer progression [61,62]. In various cancers, including breast, prostate, pancreatic, and liver cancers, B-NGF has been implicated in tumor initiation, development, and metastasis [62–65]. These findings contradict our conclusion that B-NGF acts as a protective factor for MZL. However, most research on B-NGF has primarily focused on solid tumors, with little attention given to its role in hematological malignancies. The effects of B-NGF on hematological tumors remain largely unexplored. While B-NGF may promote the growth and metastasis of solid tumors, it likely functions as a protective factor against hematologic tumors, particularly MZL. Based on our analysis, this hypothesis requires further experimental validation to confirm these findings and elucidate the mechanisms through which B-NGF influences the pathogenesis and development of MZL.

No significant association between IFN-γ levels and the risk of MZL was observed in the forward MR analysis. However, reverse MR results suggested that the onset of MZL may reduce IFN-γ levels. After accounting for potential confounding factors, heterogeneity, and pleiotropy, our bidirectional MR analysis indicates that changes in IFN-γ levels are likely a consequence of MZL, positioning IFN-γ as a potential downstream effector molecule. MZL may contribute to tumor progression by downregulating IFN-γ expression. IFN-γ is a protein consisting of two antiparallel polypeptide chains [66]. In innate immunity, IFN-γ production is primarily controlled by natural killer and natural killer T cells, whereas in adaptive immunity, CD8^+^ and CD4^+^ T cells are the main producers of this cytokine [67]. The biological effects of IFN-γ are thought to inhibit tumor progression through various pathways. IFN-γ has been shown to activate the JAK-STAT1-caspase signaling pathway, promoting tumor cell apoptosis and inhibiting tumor growth [68–71]. Liu et al. [72] reported that IFN-γ indirectly enhances tumor immunity by inhibiting fatty acid synthesis and suppressing immunosuppressive M2-like tumor-associated macrophages (TAMs). Additionally, IFN-γ interacts with various cytokines in the tumor microenvironment (TME), inducing tumor growth arrest. For instance, IFN-γ and TNF stabilize the p16INK4a-Rb pathway, promoting tumor cell senescence and dormancy [73]. Recent studies have identified IFN-γ as a marker for immune checkpoint inhibitors [67]. Based on these findings, it is evident that IFN-γ can promote inflammatory responses through multiple mechanisms within the TME, contributing to tumor suppression and inhibiting tumor growth. Therefore, IFN-γ functions as a protective cytokine against tumors. Several studies have shown that IFN-γ expression is downregulated in the solid TME of cancer patients [74–76], suggesting its potential role as a tumor suppressor, particularly in colorectal and breast cancers [76,77]. The IFN-γ signaling pathway is crucial in mediating host responses to infection, inflammation, and anti-tumor immunity, aiding in anti-tumor defense. In hematological malignancies, IFN-γ + T cells have been shown to effectively kill tumor cells, aligning with our observations [78]. However, the interaction between IFN-γ and MZL remains unexplored. Based on the physiological mechanisms of IFN-γ, its interactions with various tumors, and our MR analysis of GWAS data, we hypothesize that IFN-γ may be a downstream effector of MZL and a potential therapeutic target. Specifically, MZL may reduce circulating IFN-γ levels, inhibiting tumor cell apoptosis and senescence-induced quiescence, while promoting immune suppression and evasion, further facilitating tumor progression. However, these conclusions are based on theoretical analysis, and further experimental validation is needed to clarify the relationship between IFN-γ and MZL and to understand its biological role in MZL development.

**Figure 6:**
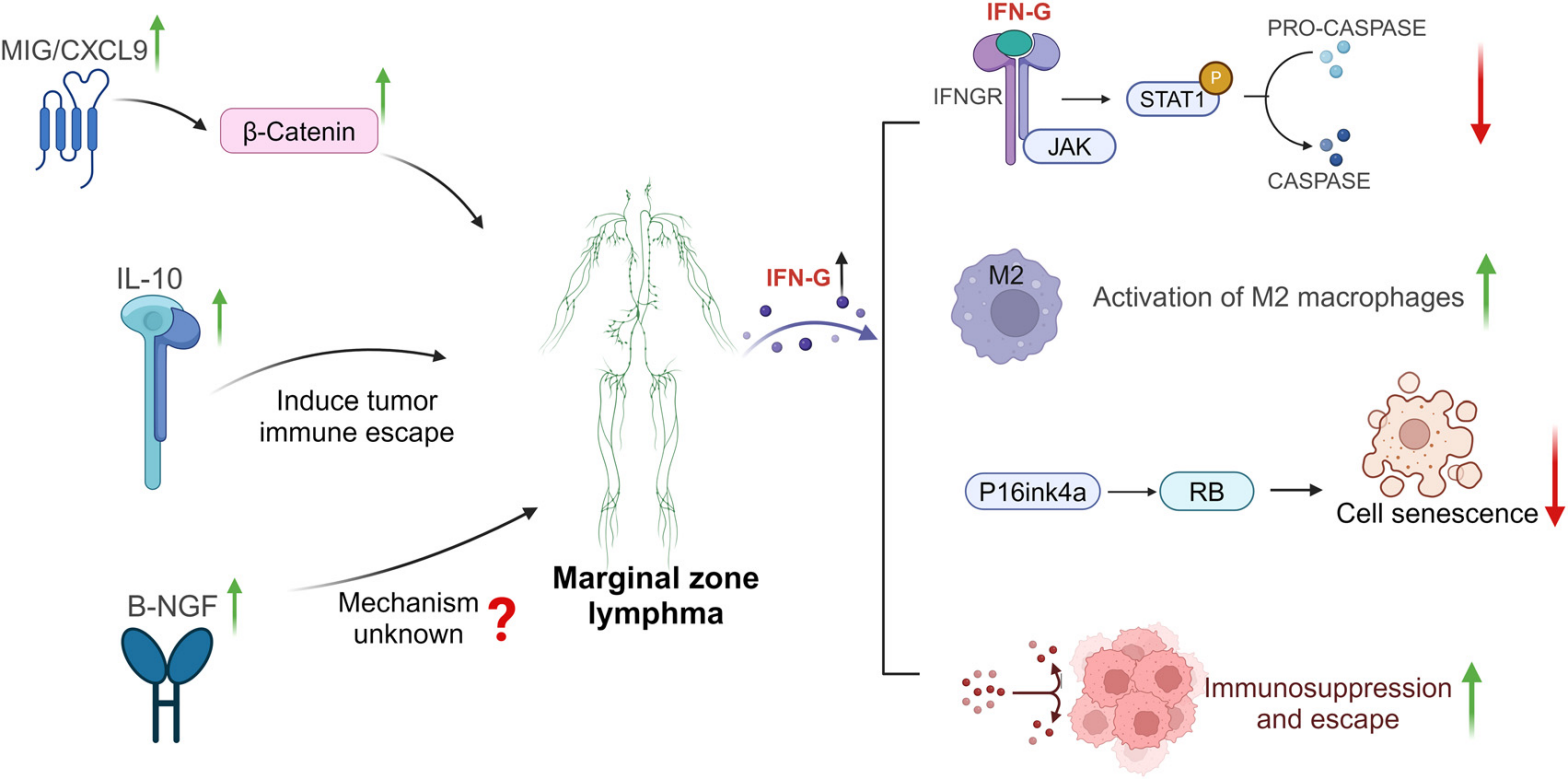
Molecular mechanisms for the causal interaction between MIG, IL10, B-NGF, IFN-G, and MZL: MIG/CXCL9 may promote MZL progression by upregulating β-catenin, while IL-10 likely facilitates tumor immune evasion, contributing to MZL development. The molecular mechanism through which B-NGF inhibits MZL progression remains unclear. MZL may reduce IFN-γ levels, potentially suppressing tumor cell apoptosis by inhibiting the JAK-STAT1-caspase signaling pathway. This reduction could enhance the activity of immunosuppressive M2-like TAMs and weaken the synergistic effect of TNF. Furthermore, through the p16INK4a-Rb pathway, it may inhibit tumor cell senescence and dormancy, leading to immune suppression and evasion, thereby promoting MZL progression.

Based on the analysis results, we propose that elevated levels of MIG and IL-10 and reduced levels of B-NGF and IFN-γ may serve as potential biomarkers for diagnosing MZL. The interaction between MIG, IL-10, B-NGF, IFN-γ, and MZL, along with the proposed mechanisms of action, is summarized in Figure 6. It is important to note that distinguishing pulmonary and gastric MZL from lung and gastric cancer can be clinically challenging. Our previous research identified high circulating levels of IL-1RA as a diagnostic marker for lung adenocarcinoma. Elevated IL-10, IL-13, and TERT levels and reduced MCP-1/MCAF levels are associated with lung squamous cell carcinoma. High levels of SDF-1α and B-NGF are indicative of small-cell lung cancer [46]. The study by Chen et al. suggests that elevated levels of circulating IL-16 and reduced levels of TRAIL serve as diagnostic markers for gastric cancer [79]. Inflammatory cytokine-related biomarkers for MZL, lung, and gastric cancer differ, highlighting their potential as auxiliary diagnostic tools to distinguish MZL from these cancers. Our findings also suggest that inflammatory cytokines could aid in differentiating MZL from other non-Hodgkin lymphoma subtypes. Previous studies identified high levels of MIG, MIP1A, PDGFbb, and low levels of IL-13 as diagnostic markers for DLBCL [21], which differ from those associated with MZL. These differences lead to the hypothesis that inflammatory cytokines could serve as supplementary references to distinguish between MZL and DLBCL. However, these conclusions are based on theoretical analysis, and further clinical trials are needed to validate whether inflammatory cytokine characteristics can serve as diagnostic standards for MZL. Incorporating these markers into clinical practice could improve the accuracy of MZL diagnosis.

With the advancement of immunotherapy, cytokine antibodies have increasingly been used in clinical and scientific studies. Recent animal research has shown that IL-10 antibodies can alleviate immune suppression and significantly enhance anti-tumor immunity in cancers such as melanoma and cervical cancer [80,81]. Building on these findings, we aim to explore whether elevated levels of MIG and IL-10, identified as risk factors for MZL, could be targeted therapeutically. Specifically, we seek to determine whether blocking the biological functions of MIG and IL-10 with specific antibodies can inhibit MZL progression. However, no clinical trials or treatments targeting these cytokines have been conducted for non-Hodgkin’s lymphoma. We expect that MIG and IL-10 antibodies could offer novel therapeutic strategies for MZL, potentially improving treatment efficacy.

However, several limitations of this study should be recognized. First, the data were derived from two large public GWAS, but the lack of detailed demographic and clinical information limited our ability to perform subgroup analyses. Additionally, the MZL dataset contained only 256 MZL cases and 345,118 healthy controls. Given the rarity of MZL, the sample size imbalance between cases and controls may impact the internal and external consistency of our MR analysis. Although we prioritized the use of IVW analysis, known for its stability, and employed multiple statistical strategies to ensure robustness, the data imbalance remains a potential limitation. Future studies should explore more robust data processing methods or incorporate external controls to further validate our conclusions. Second, we utilized a significance threshold of *p* < 5 × 10⁻⁶ for SNPs correlated with the 41 inflammatory biomarkers and *p* < 5 × 10^−5^ for the MZL SNPs. The scarcity of genome-wide significant SNPs at the more stringent *p* < 5 × 10⁻⁸ threshold limited further MR analyses. While the adjustment remains within MR principles, it introduces some risk by potentially including weak IVs, which could compromise causal inference robustness. However, multiple methods were applied to strengthen the reliability of our results. To address the limitations of this adjustment, we plan to expand the sample size in future studies to improve the robustness and reliability of our research. Third, we relied on the IVW method for data analysis and interpretation. Although results from MR-Egger, weighted average, simple, and weighted models did not reach high significance, the IVW method’s statistical power and the consistent direction of *β* values support the validity of our findings. However, due to the rarity of MZL, which accounts for about 5% of B-cell lymphoma cases, obtaining a sufficient number of MZL samples was challenging. Consequently, we were unable to validate our conclusions experimentally. In future studies, we aim to collect adequate blood and tissue samples from MZL patients and use ELISA, qPCR, and immunohistochemistry to detect and compare the levels of MIG, IL-10, B-NGF, and IFN-γ in these samples versus healthy controls. These experiments will help test our hypotheses regarding the underlying molecular mechanisms and strengthen the scientific rigor of our conclusions. Lastly, the GWAS data used in this study were exclusively from European populations, limiting the generalizability of the findings to other racial and ethnic groups. Genetic variation varies significantly among populations, and data from European populations may not accurately reflect the genetic landscape of global or non-European populations. Furthermore, the genetic structure of European populations is closely linked with specific environmental factors and lifestyle patterns, which may influence the study’s outcomes. A genetic variant associated with a disease in European populations may show no or weak associations in other populations, potentially reducing the external validity of our results. Additionally, the genetic polymorphisms observed in European populations may not fully capture the entire spectrum of genetic effects, as some variants may be rare or absent in these populations. Even within Europe, significant genetic heterogeneity exists across countries, which can influence study outcomes. Thus, caution is necessary when extrapolating these findings to non-European populations. Future research should include diverse ethnic groups and more comprehensive clinical data to validate these findings. Integrating basic experimental and clinical studies will help assess the relevance and feasibility of these results in clinical practice.

## 5 Conclusion

Using a two-sample two-way MR approach, we found that elevated levels of IL-10 and MIG are associated with an increased risk of MZL, while genetically predicted higher levels of B-NGF may act as a protective factor. The data also suggest that IFN-γ is likely a downstream molecule in MZL pathogenesis and its reduction due to MZL may further promote disease progression. No significant associations were observed between other inflammatory biomarkers and MZL. MIG, IL-10, B-NGF, and IFN-γ represent potential biomarkers for MZL, offering new and reliable strategies for its clinical diagnosis and treatment.

## Supplementary Material

Supplementary Figure

Supplementary Table 1

Supplementary Table 2

Supplementary Table 3

Supplementary Table 4
