# Supplementary material for: Exploring causal relationship between 41 inflammatory cytokines and marginal zone lymphoma: A bidirectional Mendelian randomization study
Source: Open Med (Wars). 2025 Apr 15;20(1):20251171. doi: 10.1515/med-2025-1171 (PMC12032980; doi:10.1515/med-2025-1171)
Supplement: Supplementary Figure [file med-2025-1171-sm.pdf]

Supplementary material

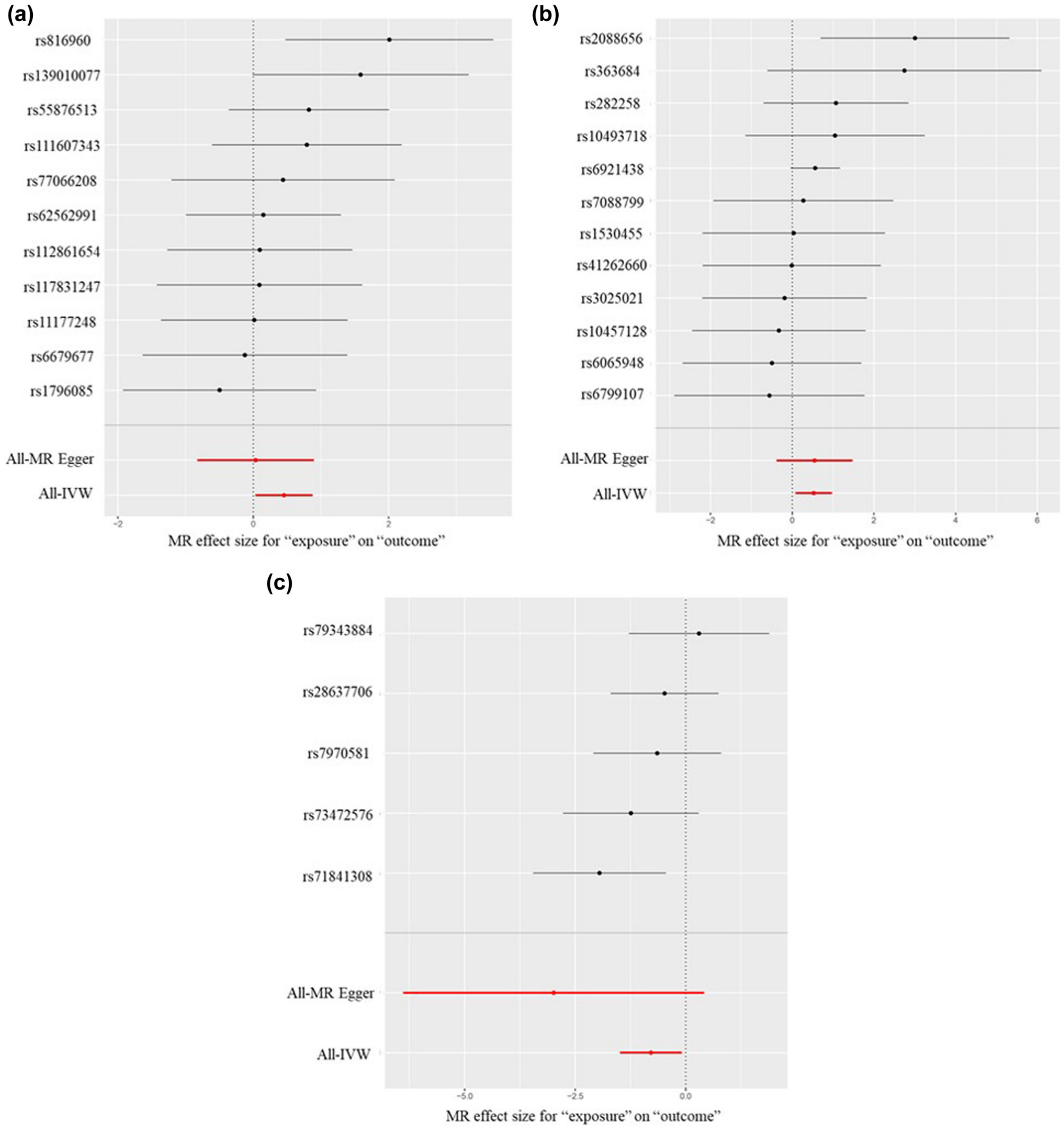

Figure S1: Forest map for positive Mendelian randomization (MR) analysis of MIG, IL-10, B-NGF and Marginal zone lymphoma: (a) Funnel plot of forward Mendelian randomization (MR) analysis of the effects of MIG on marginal zone lymphoma; (b) Funnel plot of forward Mendelian randomization (MR) analysis of the effects of IL-10 on marginal zone lymphoma; (c) Funnel plot of forward Mendelian randomization (MR) analysis of the effects of B-NGF on marginal zone lymphoma.

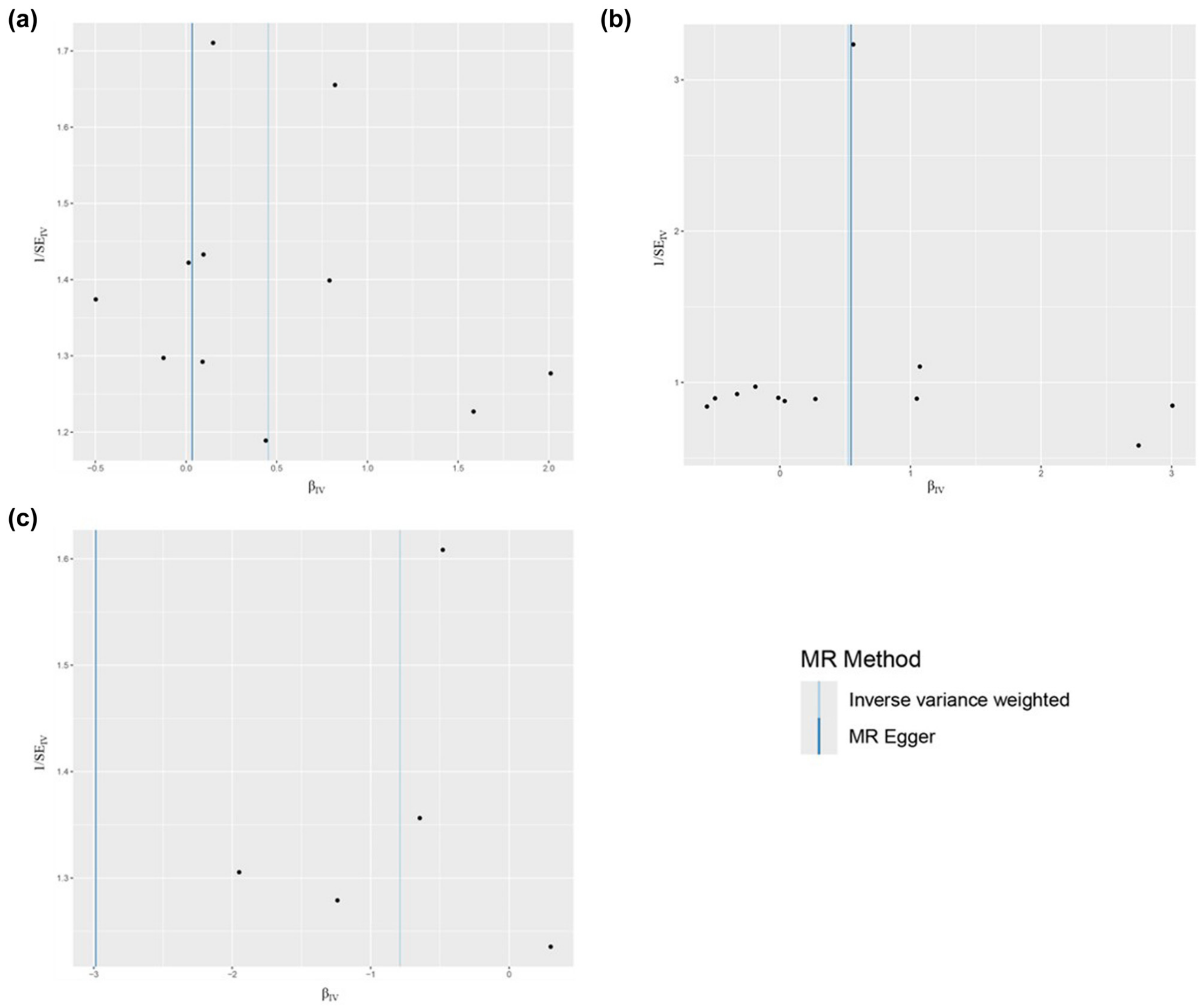

Figure S2: Funnel plot for positive Mendelian randomization (MR) analysis of MIG, IL-10, B-NGF and Marginal zone lymphoma: (a) Funnel plot of forward Mendelian randomization (MR) analysis of the effects of MIG on marginal zone lymphoma; (b) Funnel plot of forward Mendelian randomization (MR) analysis of the effects of IL-10 on marginal zone lymphoma; (c) Funnel plot of forward Mendelian randomization (MR) analysis of the effects of B-NGF on marginal zone lymphoma.

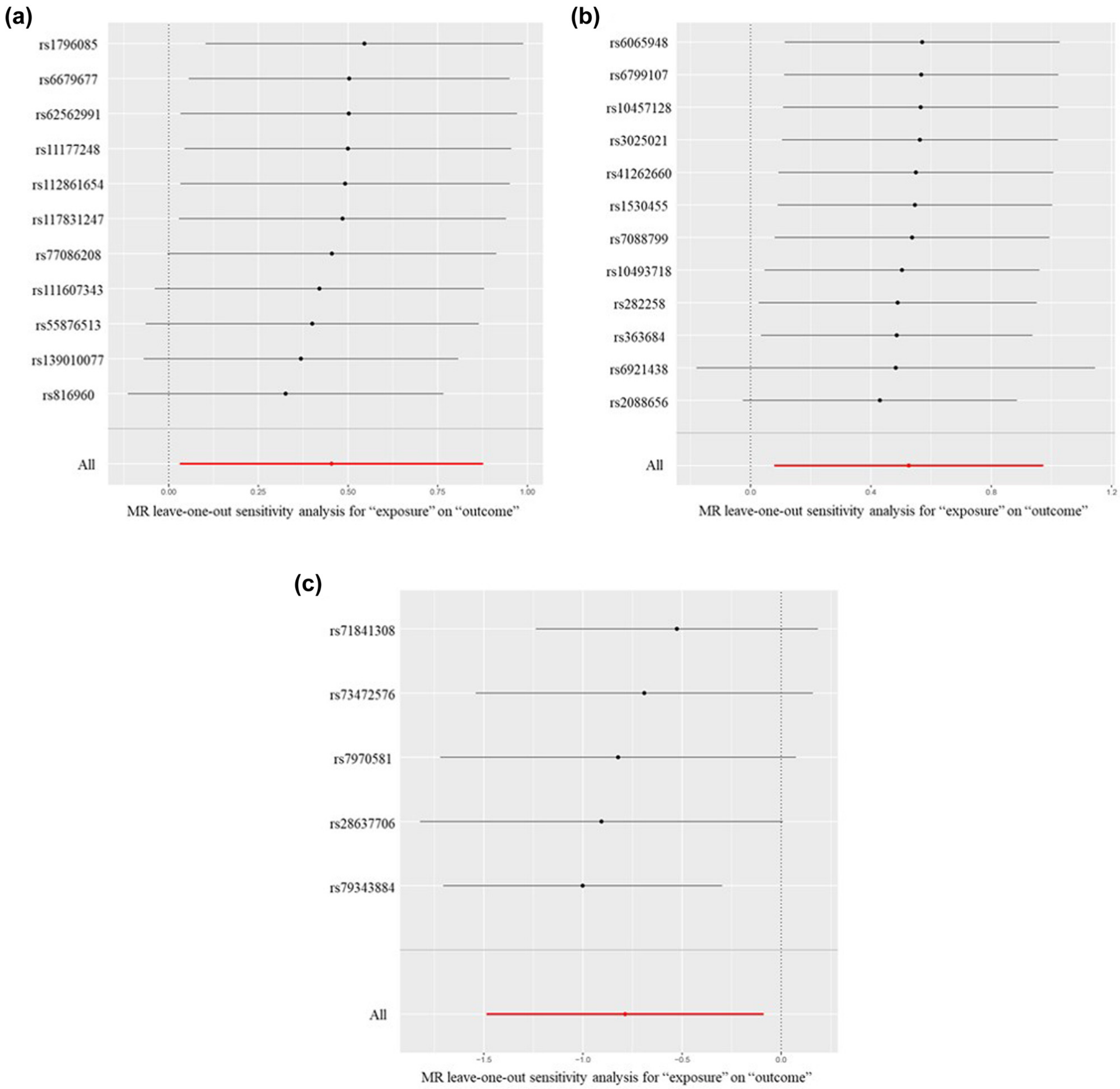

Figure S3: Leave-one-out analyses for positive Mendelian randomization (MR) analysis of MIG, IL-10, B-NGF and Marginal zone lymphoma: (a) Funnel plot of forward Mendelian randomization (MR) analysis of the effects of MIG on marginal zone lymphoma; (b) Funnel plot of forward Mendelian randomization (MR) analysis of the effects of IL-10 on marginal zone lymphoma; (c) Funnel plot of forward Mendelian randomization (MR) analysis of the effects of B-NGF on marginal zone lymphoma.
